# Social preferences under chronic stress

**DOI:** 10.1371/journal.pone.0199528

**Published:** 2018-07-18

**Authors:** Smarandita Ceccato, Sara E. Kettner, Brigitte M. Kudielka, Christiane Schwieren, Andreas Voss

**Affiliations:** 1 Universität Heidelberg, Alfred Weber Institute of Economics, Heidelberg, Germany; 2 ConPolicy – Institute for Consumer Policy, Berlin, Germany; 3 Universität Regensburg, Department of Medical Psychology, Psychological Diagnostics and Research Methodology, Regensburg, Germany; 4 Universität Heidelberg, Institute of Psychology, Heidelberg, Germany; Universidad Loyola Andalucia, SPAIN

## Abstract

Even though chronic stress is a pervasive problem in contemporary societies and is known to potentially precede both adverse psychological as well as physiological conditions, its effects on decision making have not been systematically investigated. In this paper, we focus on the relation between self-reported chronic stress and self-reported as well as behaviorally shown social preferences. We measured chronic stress with the Trier Inventory for Chronic Stress. To determine social preferences, participants played a double anonymous dictator game. In order to control for the robustness of social preferences we employed a 2x2x2x2 design where we manipulated four variables: the frame (Give to Recipient vs. Take from Recipient), the decision maker’s gender (Female vs. Male), the recipient’s gender (Female vs. Male), and the nature of the reward (Real vs. Hypothetical). Results show that perceived chronic stress is not significantly related to social preferences in monetarily rewarded dictator decisions for either gender. However, women’s displayed preferences for hypothetical rewards are negatively correlated to chronic stress levels. This indicates that higher chronic stress in women is associated with lower hypothetical transfers but not with altered actual behavior as compared to non-stressed women. For men, we do not observe such effects. Our findings suggest that, while chronic stress leaves social preferences unaffected in an incentive compatible task, it might foster what could be interpreted as a decrease in self-image promotion in women. Thus, we conclude that in a thoroughly controlled behavioral task differences in reported chronic stress do not entail differences in social preferences, but relate to variation in hypothetical decisions for women.

## Introduction

### General introduction

Chronic stress is a pervasive and growing concern in the modern world. It is generally measured using self-report measures, especially the TICS [1, 2]. Stemming from work or family conditions, it affects more and more adults, their life quality, their health-related behaviors and sleeping patterns, and it even translates onto the behavior of their children [3]. However, chronic stress not only affects the health of individuals [4], but might also affect cognitive mechanisms and decision-making processes. The vast majority of decisions are made in social contexts [5]. If chronic stress influences social decisions, this could consequently affect interpersonal relationships. Despite its importance, the cognitive effects of chronic stress have not been systematically investigated; neither at the individual level, nor in social situations. This paper is a first step towards a better understanding of the effects of chronic stress on social decisions.

Chronic stress is conceptualized as the sum of prolonged exposure to one repetitive or multiple acute stressors. The notion of acute stress in turn refers to both eustress and distress which cause a reaction in the body [6], namely the activation of the Hypothalamic-Pituitary-Adrenal (HPA) Axis and the Sympathetic-Adrenal-Medullary (SAM) Axis, measurable through, e.g., cortisol and (nor)epinephrine, respectively [7, 8]. While several biological markers have been proposed to reflect chronic stress, there is yet no robust and universal, directly validated measure (see for instance [9, 10, 11, 12], but also [13]). However, there exist several self-report-measures that have been successfully validated and employed in behavioral chronic stress research to capture perceived chronic stress [2, 14, 15, 16].

While acute stress might even promote short-term adaptation by mobilizing resources, chronic stress appears to have only damaging effects, leading, e.g., to prolonged allostatic load, the “wear-and-tear” of the body. This “wear-and-tear” is intensely studied and research has clearly established that exposure to chronic stress has both negative physiological and mental health consequences [9, 12, 17, 18, 19]. Importantly, chronic stress alters brain structures involved in cognition and decision-making [20, 21, 22, 23], consequently affecting learning and memory processes [21, 22] through, e.g., loss of hippocampal neurons [23]. Despite such findings, the effects of chronic stress on decision-making and behavior have not been consistently researched in humans, and only very few studies are yet available (for an example see [24]).

Obviously, it is important to elucidate the impact of chronic stress on individual decision-making and to try to understand whether and how it differs from the effects of acute stress. Given the social character of human beings, it is of particular significance to find out how chronic stress affects social decisions –especially as social relationships are considered helpful in situations of prolonged stress. Humans often appear to behave in a prosocial manner by nature. For instance, in the dictator game, money is regularly transferred to strangers, even in conditions of full anonymity [25]. Rand et al. show that promoting intuitive responses increases giving in dicatator games for women, but not for men [26]. A recent review suggests that acute stress might even promote such prosocial behavior, and sometimes even lead to “heroic” prosocial behaviors, especially in immediate need situations [27]. Taylor and colleagues [28] argue for a “tend-and-befriend” behavioral pattern under stress in contrast to the classical “fight-or-flight” response, where tending refers to self- and offspring protection, befriending to social networks that function as a stress buffer. This was suggested to apply in particular to women [29]. Thus, we take a first step in researching chronic stress and social decision-making in both men and women applying a task measuring social preferences with real and hypothetical payoffs. In the following, we summarize the relevant literature that motivates our experimental design.

### Literature review

Until now, research on stress and social decision-making is rather scarce. One robust finding regarding the relationship between stress and social interaction is that positive social interactions before (or during) stress exposure lead to reduced stress reactivity [30, 31, 32, 33, 34, 35, 36]. Also, initial evidence on social decision-making shows that acute stress potentially promotes prosocial behavior in men [37] and temporarily increases altruistic punishment but decreases donations in men [38]. Von Dawans and colleagues [37] used an acute laboratory stress paradigm and behavioral economic tasks to measure risk taking, trust, trustworthiness, sharing, and punishment towards an anonymous recipient. They observed that exposure to acute psychosocial stress increases non-strategic sharing, trust and trustworthiness, but not punishment or financial risk taking. Vinkers and colleagues [38] tested both strategic and non-strategic sharing, either immediately, or 75 minutes after the induction of psychosocial stress. They uncovered that strategic sharing is affected only directly after stress exposure while non-strategic sharing is generally reduced after stress, independent of time. These two experiments, displaying somewhat opposing results, included only male participants. Much stress research focuses only on men because female menstrual cycle phases and oral contraceptive intake mediate HPA axis reactivity and make control in experiments including females more difficult [39, 40]. Thus, it remains open if and how such results generalize to females. Leder et al. [41] study beauty contest games under stress and find that it generally impairs strategic reasoning.

We chose a design where both genders are studied in same and mixed gender pairs because there is strong evidence that the sexes’ stress systems and responses differ ([11, 42], their decisions in economic tasks under acute stress differ [43, 44, 45, 46, 47, 48], interactive decisions are more competitive within the same gender [49], and interaction mechanisms appear to have evolved differently for men and women. It was argued that men, as resource gatherers, might display more competitive, “fight-or-flight” responses to stress while women, as nurturers and caregivers, are more cooperative, displaying “tend-and-befriend” behaviors, in order to assure simultaneous self- and offspring-survival [28]. Thus, we include gender-pairing-related control factors, but also different frames as control variables in our design, as the existing studies described before indicate that context factors might play an important role.

Unlike acute stress and despite its pervasiveness, chronic stress has not received as much attention in behavioral research. The limited experimental research on this topic includes, to our knowledge, one pharmacological study showing that cortisol administration across eight days changes financial risk attitudes [50] and one behavioral study showing a positive correlation between self-reported stress and risk taking [24]. A recent review by Buchanan & Preston [27] suggests possible mechanisms underlying prosocial and altruistic behaviors under stress and defines very clear and specific conditions for these behaviors, mainly saliency of the recipient’s need and interactional effects. Upon these premises and considering the postulated embedded cooperation between humans in threatening times, we set out to investigate whether chronic stress modifies social preferences *per se*, independent of direct interaction with the recipient, the salience and urgency of the recipient’s need, the kinship, or the degree of danger the recipient is in. We consider that the best setting for this endeavor is a non-cooperative, interactive but socially distant context as a double anonymous dictator game where one expects participants to reveal true preferences, have no reputational concerns, and be subject to no experimenter demand effects [51, 52].

Further, we aim to increase the robustness of our results by controlling for the variation of gender-driven decisions and gender pairing, endowment ownership and reward natures. Endowment ownership is framed as either a *Give* or a *Take* treatment. Experimental variation of the reward nature (hypothetical versus real choices) helps to detect a potential hypothetical bias, i.e., when decisions in hypothetical choices are overly socially desirable as compared to those in choices involving real monetary outcomes. There is accumulating evidence showing an optimistic bias in hypothetical choices [53, 54, 55, 56], even though some studies do not find this effect in manifest behavior [57, 58] or at the neural activation level [59]. Highly relevant for our design is the fact that the hypothetical bias appears to be gender specific [59, 60] (but see also [61]) and that framing matters in this type of decisions, since a frame change (i.e., from giving to taking from an endowment) reverses the bias into a “real” bias, i.e., optimistic bias in choices involving real monetary stakes [62]. Our hypotheses relate essentially to gender and reward nature, while we use framing and gender pairing as control variables.

## Experimental design and procedure

### Experimental procedure

We set out to investigate the relationship between perceived chronic stress and social preferences in a 2x2x2x2 double anonymous dictator game [63], where neither the experimenters nor the other participants could link the decision to a participant’s identity. The four manipulated variables are: the gender of the sender (Female or Male), the gender of the recipient (Female or Male), the frame (Give to Recipient or Take from Recipient), and the nature of the reward (Real € or Hypothetical €). Below, we briefly describe the procedure. More details on the original design can be found in Kettner & Waichman [64].

Upon arrival at the experimental laboratory, participants received their show-up fee. We used two identical experimental rooms. In one room, we elicited the decisions of the dictators playing with real money. At the same time, in the other room we elicited hypothetical dictator decisions. Importantly, none of the groups was informed about the task taking place in the other experimental room. After eliciting decisions, the hypothetical dictators received the envelopes containing the decisions of the dictators playing with real money and vice versa. Demographics and psychological questionnaires were completed after the task to avoid priming or suggesting a certain type of behavior. This might make it however more probable that participants respond in a specific way to the questionnaires as to “justify” their behaviour in the task. Group assignment was randomly predetermined by numbers and letters drawn privately by participants; numbers pertained to the first experimental room, letters to the second. In the sessions where both real and hypothetical dictators were of the same sex, participants drew either a letter or a number from a common container that randomly assigned them to a designated seat in one of the two experimental rooms. In the sessions where the real and the hypothetical dictators were of opposite sex, each of the genders was directed to one of the two experimental rooms and randomly drew their seat at the entrance. Each of the two experimental rooms was supervised by both a female and a male experimenter to avoid experimenter gender biasing decisions [65].

Once all participants were seated, the experiment proceeded (for full procedures, see S1 and S2 Text). In the private cubicle of each participant, two envelopes were arranged on the table. The leftmost envelope contained the endowment. In the real money treatment this was five euros (ten fifty cent coins) and ten metal washers to preserve anonymity by mimicking the fifty cent coins in the envelopes sent to receivers in case dictators decided to keep all the euro coins for themselves (similar to Hoffman et al. [51]). In the hypothetical treatments, the envelope contained a slip of paper explaining that they had five hypothetical euros. In the Give to Recipient treatments, the leftmost envelope was labeled “Your Personal Envelope” while in the Take from Recipient treatments the rightmost envelope carried this label. The rightmost envelope was always the empty envelope. For the real money treatments dictators had to put in each of the envelopes precisely ten metal pieces (either fifty cent coins or metal washers) while for the hypothetical treatments dictators had to put in the envelopes a slip of paper containing their hypothetical transfer (from zero to five euros in increments of fifty cents). In the Give to Recipient treatments the rightmost envelope was labeled “Other [Female/Male] Participant’s Envelope”, while in the Take from Recipient treatments this was the leftmost envelope. We ran the experiment in German, where gender is embedded in the inflection and it is thus specified automatically After dictators made their decisions and placed the envelope “Other [Female/Male] Participant’s Envelope” in a collection box, they were given the demographic and psychometric questionnaires to complete. Then, collection boxes were carried into the other rooms and envelopes were given to the recipients, who opened them and counted the contents. The research assistants registered the contents on a list, privately, for each recipient.

### Chronic stress measure

We measured perceived chronic stress with the Trier Inventory for Chronic Stress [1, 2], a validated German questionnaire that requires a 10–15 minutes completion time [66]. The TICS comprises 57 items answerable on a 5-point Likert scale (‘‘never” to “very often”) and reflects the participant’s experiences within the last three months. It assesses chronic stress by nine subscales, namely “excessive workload”, “excessive social demand”, “pressure to be successful”, “dissatisfaction at work”, “mental overload at work”, “lack of social recognition”, “social tensions”, “social isolation”, and “chronic anxiety”.

We ran our experiment in thirty-three sessions as close as possible after university exam periods in February, April, and June 2013. Thus, we counted on exam preparation and exam taking as a real-life (“natural”) chronic stressor. Since this stress condition is more limited in time compared to the period reflected originally in the TICS, we adapted the questionnaires’ time reference to the last month only. Reliability analysis shows that this modified version is as reliable as the original version (Inter-item reliability analysis in S2 Table). To determine the link between chronic stress and social preferences while avoiding multiple testing in an already complex study design, we followed the scoring procedure from Schwabe and colleagues [66] computing a total chronic stress score by summing up the 57 items. Statistical parameters are reported, along with TICS scores’ descriptives, in S1 Table. The resulting variable was either used as a continuous variable in correlational and regression analyses, or was used to perform a median cut obtaining higher chronic stress versus lower chronic stress subsamples.

### Control variables

In order to control for possible confounds, we additionally collected demographic and psychometric data. Demographic controls include age, training in economics measured by a dummy indicating the completion of a game theory class, marital status, income, and altruistic history, i.e., charitable donations made in the last year. We further obtained information regarding variables that might interfere with chronic stress: prescription medication for psychiatric conditions, acute stress, symptoms of depression and anxiety. To measure acute stress and emotional activation we used a Visual Analogue Scale (from 0 to 100). To control for depression and anxiety, we used the validated German version of the HADS—Hospital Anxiety and Depression Scale [67].

### Participants

The experiment was conducted at the AWI Laboratory, University of Heidelberg, Germany. The initial study sample consisted of 376 adults. They were inexperienced participants recruited from the participant pool via ORSEE [68]. Our final analysis sample includes 348 participants. Sixteen participants had to be excluded because of taking prescription medication for psychiatric conditions, six participants were excluded due to incomplete chronic stress questionnaires (According to the instructions delineated in the TICS manual [2]. Two participants misunderstood the task, and five participants were of senior age, deviating from the age average and thus characteristics of our target population (age average 46.8 versus 22.7 in the remaining sample). The mean age of our final sample was 22.7 years and ranged from 18 to 33 years with 50.3% women and 49.7% men. Further sample properties, including basic data on transfers, are detailed in a working paper by Kettner & Ceccato [69].

## Hypotheses

From the literature review, two main hypotheses follow:

Hypothesis 1: Chronic stress is positively related to real money transfers in the dictator game; this result is robust to frame and gender-pairing manipulations.
Hypothesis 2: Since there is no reason to assume that real and hypothetical transfers differ in one specific direction, we expect that chronic stress is also positively related to hypothetical money transfers in the dictator game; this result is robust to frame and gender-pairing manipulations. We further expect a stronger hypothetical bias in females.

## Results

In the following, we first perform hypotheses testing. In section 4.1 we first present mean transfers and compare transfers between chronic stress levels and for the different treatments. In section 4.2 we check the robustness of the results by performing supplementary regression analyses.

### Chronic stress is only related to (women’s) hypothetical transfers

Table 1 shows the average transfers for the different reward natures. Subsample sizes, demographic information and transfer values are provided, contrasting higher versus lower stress groups (median split). We divide the transfers into real money transfers (Real) and hypothetical money transfers (Hypothetical), as these two conditions are fundamentally different [69], but we also pool them for comparison later. For each reward nature, we report average transfers pooled across genders first. Then, gender-specific transfers in conjunction with stress levels are analyzed. Table 2 presents Mann-Whitney U Tests comparing transfers between higher stressed versus lower stressed participants for both genders and frames in Real, Hypothetical, and Real vs. Hypothetical conditions.

**Table 1:** Average transfers of higher stressed (HS) and lower stressed (LS) participants in the real and hypothetical conditions.

Real							Hypothetical					
	Mixed HS	Mixed LS	Female HS	Female LS	Male HS	Male LS	Mixed HS	Mixed LS	Female HS	Female LS	Male HS	Male LS
Percentage transferred	19.88 (24.17)	21.49 (21.74)	24.29 (25.90)	22.86 (21.33)	13.71 (20.30)	20.51 (22.16)	26.36 (23.45)	34.00 (26.56)	31.67 (22.63)	43.33 (25.30)	20.00 (23.09)	25.38 (25.01)
N	84	101	49	42	35	59	88	75	48	36	40	39
Age	22.66 (2.96)	22.83 (2.25)	22.58 (3.17)	22.67 (2.22)	22.77 (2.70)	22.95 (2.29)	22.68 (2.71)	22.75 (2.35)	22.46 (2.60)	23.17 (2.57)	22.95 (2.86)	22.36 (2.08)

Note: The table displays mean values (standard deviations in parentheses). The percentage transferred is calculated from the initial endowment of € 5.

**Table 2:** Mann-Whitney U Tests comparing transfers between lower stress and higher stress participants in the real, hypothetical and real vs. hypothetical conditions.

Real		Hypothetical		Real vs. Hypothetical	
LS- vs. HS-	p = .41	LS- vs. HS-	p = .08 (+)	R----- vs. H-----	p < .001 (***)
FLS- vs. FHS-	p = .88	FLS- vs. FHS-	p = .02 (*)	R--LS- vs. H--LS-	p = .002 (**)
MLS- vs. MHS-	p = .17	MLS- vs. MHS-	p = .36	R--HS- vs. H--HS-	p = .04 (*)
				R-F--- vs. H-F---	p < .001 (***)
				R-M--- vs. H-M---	p = .18
				R-FLS- vs. H-FLS-	p < .001 (***)
				R-FHS- vs. H-FHS-	p = .08 (+)
				R-MLS- vs. H-MLS-	p = .33
				R-MHS- vs. H-MHS-	p = .23

Note: p-values are rounded to two decimals and significant results are flagged with (+) for trends where p < .10, with (*) for p < .05, with (**) for p < .01 and with (***) for p < .001. LS vs. HS indicate low vs. high stress. F/M indicates the gender of the sender.

Table 1 shows that transfers with hypothetical rewards were higher than with real money and in Table 2 it can be seen that this difference is statistically significant (p < .001). Participants with stress scores above the median transferred on average 0.98 € (19.88% of the initial endowment) when the task was incentive compatible, and 1.32 € (26.36% of the initial endowment) when the transfer was hypothetical. This difference was slightly stronger for lower stressed participants: they transferred 1.09 € (21.49% of the initial endowment) in the Real transfer condition and 1.70 € (34.00% of the initial endowment) in the Hypothetical transfer condition (p = .001). Importantly, this result seems to be driven by females, irrespective of their chronic stress level (see Table 2). Women in the lower stress group claimed to transfer almost double 2.17 € (43.33% of the initial endowment) in the Hypothetical transfer condition compared to what they transferred in the Real condition, namely 1.14 € (22.86% of the initial endowment, p < .001).

Finally, as indicated by transfer percentages in Table 1, women’s behavior differs from that of men in general, in that they always transfer more. This effect is marginally significant for decisions involving actual money (p = .09) and significant for decisions involving pretended transfers (p < .001). It is in line with recent findings in the literature showing that women tend to be more altruistic than men in dictator games [26], [70].

When we compared different transfers within the same reward nature condition, we discovered that it was only in the Hypothetical condition where participants with higher chronic stress levels claimed to behave differently (transferring less) compared to participants with lower chronic stress levels (p = .08). On a descriptive level, we observed the same pattern for men in the Real condition, but this remained non-significant. In sum, we so far observed that real transfers did not significantly differ between subjects reporting higher versus lower stress levels while hypothetical transfers differed and this effect seems to be driven mainly by women’s claims. Generally, hypothetical transfers were significantly higher than those involving actual money.

**Fig 1:**
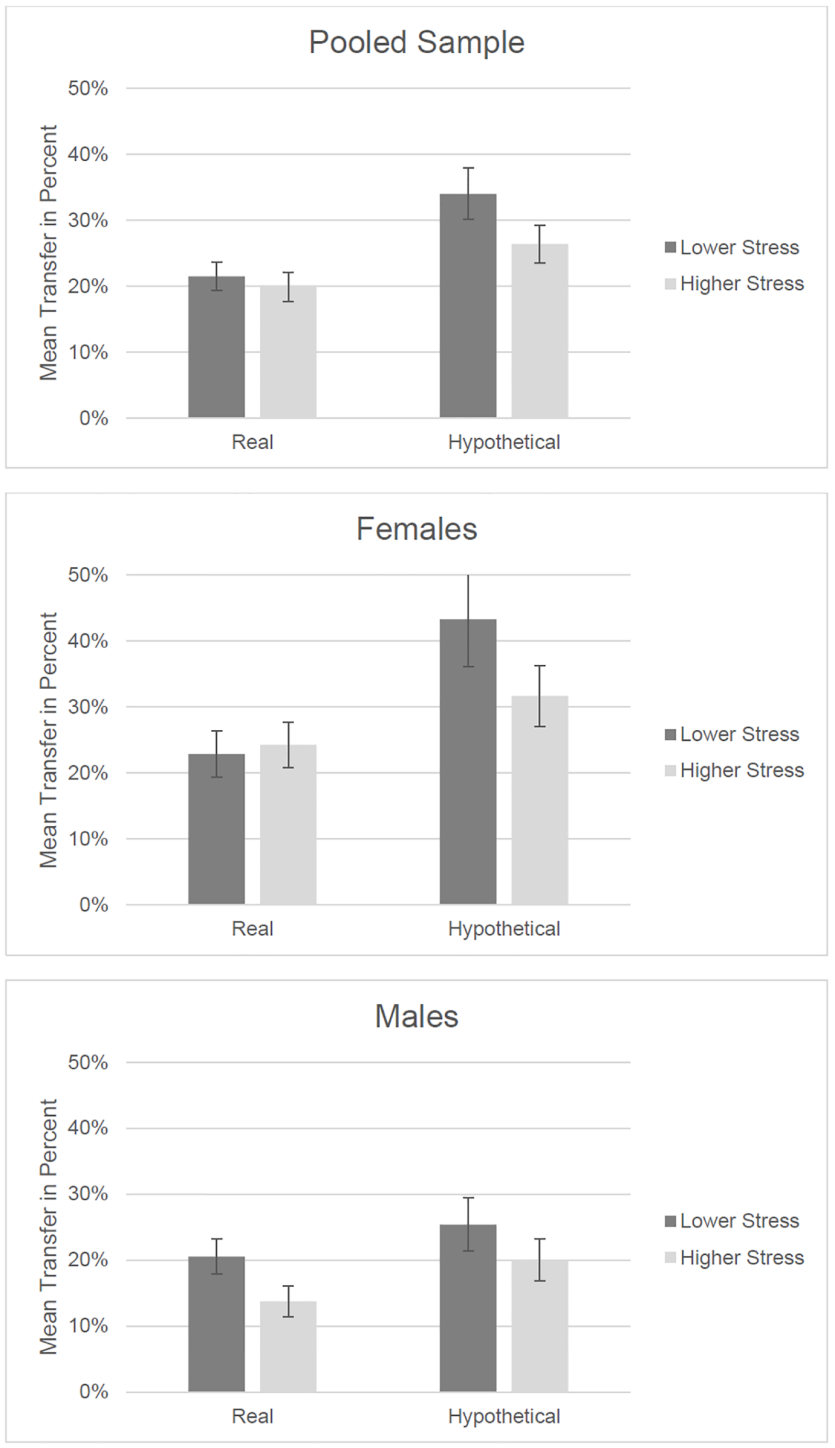
Mean transfers (in percentage) for real and hypothetical reward natures, for the pooled sample (both men and women) and for females and males, respectively.

In Fig 1 results are depicted graphically. As can be seen in the upper left panel of Fig 1, both real and hypothetical money transfers are lower when chronic stress levels are higher. However, the difference is significant only when decisions do not involve real money. The upper right panel, splitting the data by gender, shows that, for men, higher chronic stress is associated with lower transfers, irrespective of reward nature. For women, the nature of the reward changes the direction of the difference: in the Real condition, the difference is not significant, but positive (i.e., higher transfer for those reporting high chronic stress), whereas in the Hypothetical condition, lower transferred amounts correlate with higher reported chronic stress.

The results so far can be summarized as follows:

Result 1.1: Chronic stress does not significantly correlate with incentive compatible (Real) transfers.
Result 1.2: Chronic stress is significantly and negatively related to hypothetical transfers.

Thus, so far we seem to have to refute both hypotheses. So far, however, we have only done pair-wise testing, but as there are many comparisons involved, we have to move towards testing our hypotheses more rigorously in a regression analysis, involving also the control variables we have collected. This is what we do in the following section.

### Regression analyses

We now turn to regression analyses including also all control variables to test for the robustness of the results. We run one overall model with the main explanatory variables, and later add more controls (Table 3). The first model explains transfers with the continuous score of the TICS alone. The second model adds the main variables of our hypotheses: the sender’s gender (1 = Female and 0 = Male), and the condition (real vs. hypothetical). The third model adds the interactions between gender and the TICS-score and between Gender and the condition (real vs. hypothetical) and the three-way interaction with gender, TICS score and condition. Model (4) adds all other controls: gender of the recipient, frame, acute stress, depression, anxiety levels, age, game theory background knowledge (dummy variable), marital status ((1 = Single and 0 = In a Relationship), income level, and donation history (1 = Donation history and 0 = No donation history). Power was analyzed using G*Power 3.1 [71]. For a multiple regression, power of the present study to detect medium sized effects (f^2^ = 0.15) is excellent (1-β>.99). For small effects (f^2^ = 0.02) power is lower but still acceptable (1-β = 0.75).

**Table 3:** Robust OLS (standard errors in parentheses).

	(1)	(2)	(3)	(4)
	Transfer in Percent	Transfer in Percent	Transfer in Percent	Transfer in Percent
TICS	-0.08* (0.04)	-0.10* (0.04)	-0.11* (0.05)	-0.11 (0.07)
Gender Sender (0 = male, 1 = female)		9.25*** (2.43)	13.96*** (3.55)	14.23***(3.71)
Condition (0 = Hypo.,1 = Real)		-8.54*** (2.43)	-4.51 (3.45)	-2.32 (3.60)
Gender x TICS			-0.09 (0.10)	-0.07 (0.10)
Gender x Condition			-8.75^+^ (4.86)	-9.81^+^ (5.00)
Gender x Condition x TICS			0.24^+^ (0.12)	0.22^+^ (0.12)
Frame (0 = loss, 1 = win)				-5.44* (2.54)
Gender Recipient				1.83 (2.51)
Game Theory (1 = yes)				-9.10*** (2.70)
Donation (1 = yes)				1.38 (2.57)
Age				0.48 (0.49)
Income				1.75 (2.51)
HADS_A				-0.04 (0.48)
HADS_D				0.29 (0.55)
AcuteStress				-0.03 (0.05)
Single				4.51 (2.81)
Constant	24.52*** (1.26)	26.38*** (2.17)	22.15*** (2.54)	8.76 (13.22)
Observations	348	348	348	343

Note:

^+^*p* < .10;

*p < .05;

***p < .001

With respect to the joint sample for both conditions, TICS shows significant negative coefficients in models (1), (2) and (3), while being only marginally significant in model (4), indicating less contributions under high stress. A highly significant effect of Gender of the sender is based on higher contributions from women. There is also a significant difference for frame, indicating a reduced (increased) contributions in the take- (loss) frame. Additionally, data revealed a highly significant negative effect of condition in model 2, suggesting that in the real condition contributions are lower than in the hypothetical condition. Most important, this effect is no longer significant, when the three-way interaction is included. To disentangle the effect of the three-way interaction, we tested the effect of stress on contributions separately in the 2x2 groups. Results revealed a slightly positive relation of stress and contributions for women in the real-reward condition (b = 0.05), and negative relations in all other groups (b = -0.20; b = -0.11; b = -.10; for female/hypothetical, male, real, and male hypothetical, respectively). This pattern suggests that only in the hypothetical condition there is a clear (negative) relationship between chronic stress and giving behavior in the dictator game, while in the real reward condition, we find an interaction with gender: While female dictators give more under stress, the relation of stress and giving behavior is reversed for man. Results from these regression analyses also hold in a Tobit regression (see S3 Table).

Results confirm that chronic stress successfully contributes to an explanation of differences in hypothetical transfers. This effect emerges for the overall sample, but is driven by women. Importantly, results are robust to all the controls we introduced.

In sum, we conclude:

Result 2.1: Chronic stress tends to increase (real) transfers for women and decrease (real) transfers for men.
Result 2.2: Chronic stress is negatively related to hypothetical transfers. This result is robust to experimental manipulations and possible confounds.

Thus, we have to refute both our hypotheses.

## Discussion

Our present data shows that self-reported chronic stress as measured by the TICS is not related to social preferences in an incentive compatible task, but is related to social preferences in a hypothetical task for women. With this, we cannot support, neither for men nor for women, the initial findings from the acute stress literature of von Dawans and colleagues [37], where acute stress exposure was positively associated with prosocial behavior (i.e., higher money transfers) in men. In contrast, we conclude that chronic stress does not relate to increased prosocial behavior. This appears to be in accordance with Vinkers and colleagues [38]. However, one should note that we are reporting on a very different condition of stress in a substantially different design.

In a laboratory social stress paradigm, von Dawans and colleagues [37] showed that men behave more prosocial after stress exposure. After inducing acute social stress through socio-evaluative threat (the TSST-G protocol- [72]), 34 non-economist males made binary choices measuring trust, trustworthiness, sharing, and punishment towards an anonymous male recipient they might have interacted with before the task, as well as financial risk taking. Von Dawans and colleagues [37] found that exposure to psychosocial stress increased sharing (higher proportion of non-zero transfers), as well as trust and trustworthiness, but did not have any effect on punishment or financial risk taking. They motivate their findings by the theories of Taylor et al. [28] and Taylor [29], accounting for a “tend-and-befriend” behavioral pattern, also among interacting men. In contrast to this finding, our result is more in line with the conclusions of Vinkers and colleagues [38]. They tested social decision-making under acute psychosocial stress in a sample of 80 male participants. Strategic sharing (the ultimatum game) and non-strategic sharing (the dictator game) were either assessed immediately after stress exposure or 75 minutes later. Then, subjects played a donation dictator game, deciding how much, if anything, of 10 endowed euros to donate to UNICEF. The results show that the effects of acute stress on strategic sharing were time-dependent while those on non-strategic donation are not time dependent. Donations in the dictator game were generally reduced in stressed participants, regardless of timing. The authors suggest that this raises the idea that acute psychosocial stress exposure does not modify reward sensitivity, but might merely decrease self-control, which is reestablished after stress effects have decayed. The design we implemented is more similar to the one described in the second experiment [38]. In accordance with these results, our data might point in the same direction, namely towards a higher self-interest (rationality) under increased stress. However, our experiment digresses from the above in several ways.

First, we looked at chronic and not acute stress. Acute stress refers to a momentary activation of the stress system while chronic stress entails long-term, repeated activation. Most literature defines this as a period of at least two weeks, but some authors propose also a minimum of one month. Furthermore, the biological reaction to chronic stress is probably more strongly dependent on personal characteristics than that to acute stress (e.g., neuroticism, trait anxiety), but, to date, chronic stress lacks a reliable physiological marker. Second, the sharing task that von Dawans et al. [37] use is a dictator game, but they only offered one of two possibilities to the decision maker: either send nothing or the fair-share, i.e., half of the initial endowment. We, in contrast, measured social preferences in a continuous manner that allows expressing prosocial attitudes in a more nuanced way. Third, as Buchanan & Preston [27] concluded, prosociality is closely connected to interacting actors as well as the urgency and the saliency of the recipient’s need. We purposefully tried to avoid low social distance by setting up a double anonymous context in order to investigate if chronic stress affects social preferences *per se*, as opposed to immediate responses to demand or need.

We also incorporated a hypothetical setting, substantiating an idea raised by Buchanan & Preston [27]. In their review, they hypothesized that stress might not change social behavior, especially if it is not a social stressor, but it may affect the capacity of individuals to focus on positive self-presentation. In our data, we observed this in particular for women. This is in line with other results indicating that women in general are more responsive to subtle social cues as to what is the appropriate behavior in a specific situation [43]. In our study, this gender specificity applies to both the general hypothetical bias and the connection between stress and behavior. One might argue that this is partially due to the fact that women report significantly higher stress levels in our experiment and in the literature in general [73]. It might, however, also be due to the fact that men simply put their money where their mouth is and also do not pretend to act differently. In contrast, women in a hypothetical situation might consider carefully how to present themselves, especially if they do have the mental capacity to do so, i.e., if they are not stressed. This, of course, cannot be tested with our data and should be analyzed in future research.

Finally, the existing literature on acute stress and social decision-making is based on cortisol levels as biological measure of acute stress while we rely on a self-report measure of chronic stress. This makes results difficult to compare, as we might be measuring very different aspects of the stress construct. The relation between perceived and reported stress on the one hand and biological activation of the stress system on the other is not as straightforward as one would expect. While studies exist that report on correlations between acute stress markers and acute stress self-reports (for instance, [38]), other studies reported on a lack of covariance between physiological and subjective stress markers (see, for instance, [37, 74, 75]). In what concerns chronic stress, matters are even more complicated since there seems not to exist any biological measure that reliably correlates with self-reported long-term stress.

Another important issue to mention is causality. While we carefully tried to control for possible other mechanisms driving our results, we only report correlational evidence, and thus cannot rule out completely that it is not chronic stress that drives our results, but some other variable that is related both to chronic stress and dictator giving behavior.

## Conclusion

We investigated the correlation between self-reported chronic stress as measured by the TICS and social preferences in a double anonymous dictator game. We manipulated the frame, the gender-pairing, and the nature of the reward. We showed that there is no significant correlation between chronic stress and social preferences for real rewards. We also showed that this lack of correlation is robust across all experimental conditions and further controls. However, women’s hypothetical social preferences are significantly negatively correlated to self-reported chronic stress levels: the higher their reported stress, the less they declare to be willing to send to an anonymous recipient.

This lower level of other-regarding intentions is independent of various possible controls and almost all experimental manipulations, except for the framing. We can only speculate on the reasons for the difference between our findings and those by von Dawans et al. [37]. What becomes clear from our results is that the relationship between perceived chronic stress and behavior warrants further research effort, as one cannot just extrapolate results on acute to chronic stress. Furthermore, it seems important to focus on potential gender specifics in stress research.

## Supporting information

S1 Text Translated instructions 1: Real rewards dictator game. (PDF)

S2 Text Translated instructions 2: Hypothetical rewards dictator game. (PDF)

S1 Fig Metal washers used to preserve anonymity. (TIF)

S1 Table Descriptive data. (PDF)

S2 Table Modified TICS is highly reliable. We tested reliability for the TICS summed value, as well as for the original screening scale for chronic stress (SSCS) and the nine facets of stress determined in the questionnaire: pressure to be successful (ERDR), lack of social recognition (MANG), chronic anxiety (SORG), excessive social demand (SOUE), social isolation (SOZI), social tensions (SOZS), excessive workload (UEBE), mental overload at work (UEFO), and dissatisfaction at work (UNZU).
(PDF)

S3 Table Relation chronic stress & transfers in incentive compatible task. (PDF)
