# Supplementary material for: Social preferences under chronic stress
Source: PLoS One. 2018 Jul 18;13(7):e0199528. doi: 10.1371/journal.pone.0199528 (PMC6051590; doi:10.1371/journal.pone.0199528)
Supplement: S1 Text — (PDF) [file pone.0199528.s001.pdf]

S1 Translated Instructions 1: Real Rewards Dictator Game

In what follows we present the translated instructions of the Give Male to Female treatment. Please note that the translation highlights the gender of both sender and recipient. In German this is not necessary since gender is embedded in the in the inflection.

General Information

Dear (male) participant,

Thank you for participating in this study on decision making. In the following, you will be informed about the rules and procedures. Every (male) participant has received the same printed instructions as you did. Please take your time and read the instructions carefully.

No communication with other (male) participants

All decisions in this study are private. Please do not communicate with the other (male) participants. Otherwise, we are forced to exclude you from the experiment and you will have to forgo your payment. If you have any questions, please raise your hand. The (male or female) experimenter will answer your question quietly.

Anonymous matching

In this study, you will be randomly matched with another (female) participant from the other room. The randomization is carried out according to the number you drew during registration at the beginning of this study. The matching will not be made public and no (male) participant can reconstruct which other (female) participant he is matched to. This experiment is completely anonymous. Your identity will not be made public and you will not receive information about the identity of the other (male) participants in this room and the other (female) participants in the other room.

General information about the decision task

Both you and the matched (female) participant received 5 € for your participation at the beginning. In addition to this, you have another 5 € which are in “Your Personal envelope”, on the table in front of you. The other matched (female) participant has nothing (0 €). You can now leave the amount you just received unchanged or reduce it, and increase the amount of the (female) participant you have been matched with.

How to make your decision

On the table in front of you, you see two envelopes: one is your personal envelope and the other envelope belongs to the other (female) participant. In order to distinguish between the envelopes, they are marked: your personal envelope is marked "YOUR PERSONAL ENVELOPE"; the envelope of the other (female) participant is marked "ENVELOPE OF OTHER (FEMALE) PARTICIPANT".

Content of the two envelopes

Your personal envelope contains a total of 20 coins, out of which ten are 50 cents coins (5 €) and ten are worthless coins (metal washers). The washers have the purpose of keeping your decision completely anonymous with respect to other persons including the (male and female) experimenters.

The envelope of the other (female) participant is empty.

Please make sure that your personal envelope contains ten 50 cents coins and ten worthless washers by emptying the contents onto the table in front of you.

Receipt 2: This receipt is only for accounting purposes. After you signed the receipt, we ask you to place it in the sealed collection box and continue with your decision. The sealed box is

used so that the (male and female) experimenters cannot see the name written on the receipt. All (male) participants in this room sign the second receipt. The (female) participants in the other room will not sign such a receipt.

The decision

After you have emptied the contents of your personal envelope on the table in front of you and signed the receipt, please put exactly ten coins/washers back in your personal envelope. Similarly, put exactly ten coins/washers into the envelope of the other (female) participant. In the appendix, we present all possible decisions (for the appendix see last page of instructions).

Completing the decision and sealing the envelopes

As soon as you have made your decision, put your personal envelope into your pocket (coat, etc.). Please seal the envelope of the other (female) participant (i.e. use the flap-tape to seal the envelope) and place it in the box located behind you, on the floor. (Important: Please do not hand the envelope to another person or to the (male or female) experimenter, but place it directly in the collection box.) After all (male) participants in this room have made their decision, a (male or female) experimenter will carry the box to the other room in which a second (male or female) experimenter will take over the box and distribute the envelopes to the assigned (female) participants. Nobody in the other room is informed about your identity.

Anonymity

We have planned the experiment in a way which guarantees your anonymity at all times.

1. Your identity is never revealed to another person.

2. The (male or female) experimenter who distributes the envelopes to the (female) participants in the other room was not present at the time you made your personal decision. He or She and the other (female) participants do not know from whom they received the envelope.

3. After the decision we will ask you to fill in an anonymous questionnaire. The questions are used for the evaluation of the study and none of your answers can be linked to your identity.

Thank you very much for your support!
