# Supplementary material for: Social preferences under chronic stress
Source: PLoS One. 2018 Jul 18;13(7):e0199528. doi: 10.1371/journal.pone.0199528 (PMC6051590; doi:10.1371/journal.pone.0199528)
Supplement: S2 Text — (PDF) [file pone.0199528.s002.pdf]

S2 Translated Instructions 2: Hypothetical Rewards Dictator Game

In what follows we present the translated instructions of the Take Female from Female treatment. Please note that the translation highlights the gender of both sender and recipient. In German this is not necessary since gender is embedded in the in the inflection.

General Information

Dear (female) participant,

Thank you for participating in this study on decision making. In the following you will be informed about the rules and procedures. Every (female) participant has received the same printed instructions as you did. Please take your time and read the instructions carefully.

No communication with other (female) participants

All decisions in this study are private. Please do not communicate with the other (female) participants. Otherwise, we are forced to exclude you from the experiment and you will have to forgo your payment. If you have any questions, please raise your hand. The (male or female) experimenter will answer your question quietly.

Anonymous matching

In this study, you will be randomly matched with another (female) participant from the other room. The randomization is carried out according to the number you drew during registration at the beginning of this study. The matching will not be made public and no (female) participant can reconstruct which other (female) participant she is matched to. This experiment is completely anonymous. Your identity will not be made public and you will not receive information about the identity of the other (female) participants in this room and the other (female) participants in the other room.

General information about the decision task

Both you and the matched (female) participant received 5 € for your participation at the beginning. In addition to this, she has another 5 hypothetical € which are in the "Other (female) Participant's envelope", on the table in front of you. You have nothing hypothetical (0 hypothetical €). You can now leave her amount unchanged or reduce it, and hypothetically increase your amount.

How to make your decision

On the table in front of you, you see two envelopes: one is your personal envelope and the other envelope belongs to the other (female) participant. In order to distinguish between the envelopes, they are marked: your personal envelope is marked "YOUR PERSONAL ENVELOPE"; the envelope of the other (female) participant is marked "ENVELOPE OF OTHER (FEMALE) PARTICIPANT".

Content of the two envelopes

The envelope the other (female) participant contains a slip of paper on which is specified that she has an additional, hypothetical 5€.

Your personal envelope is empty.

Please make sure, that the envelope of the other (female) participant contains a slip of paper on which is specified that she has an additional, hypothetical 5€ and your personal envelope contains a slip of paper specifying that your hypothetical payoff is 0€.

The decision

After you have emptied the contents of the other (female) participant's envelope on the table in front of you, please put write down how much of that hypothetical 5€ you would like to return

to her, from 0€ to 5€ in increments of 0.50€. In the appendix, we present all possible decisions (for the appendix see last page of instructions).

Completing the decision and sealing the envelopes

As soon as you have made your decision, put your personal envelope into your pocket (coat, etc.). Please seal the envelope of the other (female) participant (i.e. use the flap-tape to seal the envelope) and place it in the box located behind you, on the floor. (Important: Please do not hand the envelope to another person or to the (male or female) experimenter, but place it directly in the collection box.) After all (female) participants in this room have made their decision, a (male or female) experimenter will carry the box to the other room in which a second (male or female) experimenter will take over the box and distribute the envelopes to the assigned (female) participants. Nobody in the other room is informed about your identity.

Anonymity

We have planned the experiment in a way which guarantees your anonymity at all times.

1. Your identity is never revealed to another person.

2. The (male or female) experimenter who distributes the envelopes to the (female) participants in the other room was not present at the time you made your personal decision. He or She and the other (female) participants do not know from whom they received the envelope.

3. After the decision we will ask you to fill in an anonymous questionnaire. The questions are used for the evaluation of the study and none of your answers can be linked to your identity.

Thank you very much for your support!

You	Other (female) participant	Return to your personal envelope	Place in envelope of other (female) participant
5€	0€	10 x 50 cents coins and 0 x washers	0 x 50 cents coins and 10 x washers
4.5€	0.5€	9 x 50 cents coins and 1 x washers	1 x 50 cents coins and 9 x washers
4€	1€	8 x 50 cents coins and 2 x washers	2 x 50 cents coins and 8 x washers
3.5€	1.5€	7 x 50 cents coins and 3 x washers	3 x 50 cents coins and 7 x washers
3€	2€	6 x 50 cents coins and 4 x washers	4 x 50 cents coins and 6 x washers
2.5€	2.5€	5 x 50 cents coins and 5 x washers	5 x 50 cents coins and 5 x washers
2€	3€	4 x 50 cents coins and 6 x washers	6 x 50 cents coins and 4 x washers
1.5€	3.5€	3 x 50 cents coins and 7 x washers	7 x 50 cents coins and 3 x washers
1€	4€	2 x 50 cents coins and 8 x washers	8 x 50 cents coins and 2 x washers
0.5€	4.5€	1 x 50 cents coins and 9 x washers	9 x 50 cents coins and 1 x washers
0€	5€	0 x 50 cents coins and 10 x washers	10 x 50 cents coins and 0 x washers
