# Supplementary figures and images for: Social preferences under chronic stress
Source: PLoS One. 2018 Jul 18;13(7):e0199528. doi: 10.1371/journal.pone.0199528 (PMC6051590; doi:10.1371/journal.pone.0199528)

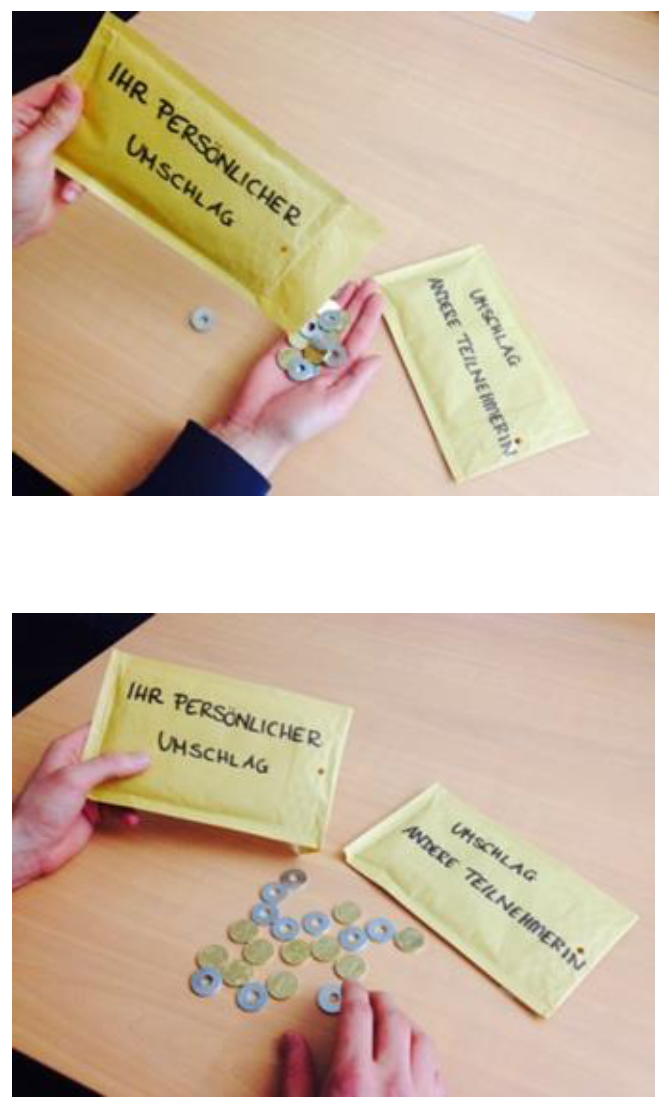

Supplement: S1 Fig — (TIF) [file pone.0199528.s003.tif]
