# Supplementary material for: Social preferences under chronic stress
Source: PLoS One. 2018 Jul 18;13(7):e0199528. doi: 10.1371/journal.pone.0199528 (PMC6051590; doi:10.1371/journal.pone.0199528)
Supplement: S1 Table — (PDF) [file pone.0199528.s004.pdf]

S1 Table: Descriptive Data

	Real Reward			
**Give**	**Male to Male**	**Male to Female**	**Female to Male**	**Female to Female**
N	24	22	25	21
Transfer in Percent	11.67	18.18	25.60	24.76
Age (in years)	23.58	22.55	22.24	22.9
Income level	1.71	1.82	1.56	1.67
Single in Percent	73.91	81.82	68	52.38
Game theory knowledge in Percent	54.17	59.09	24	28.57
Donation history in Percent	30.43	59.09	60	52.38
**Take**	**Male to Male**	**Male to Female**	**Female to Male**	**Female to Female**
N	24	24	22	23
Transfer in Percent	14.17	27.92	29.09	15.22
Age (in years)	22.88	22.5	22.57	22.83
Income level	1.67	1.75	1.64	1.70
Single in Percent	79.17	70.83	72.73	69.57
Game theory knowledge in Percent	66.67	62.5	27.27	43.48
Donation history in Percent	62.5	70.83	50	69.57

	Hypothetical Reward			
**Give**	**Male to Male**	**Male to Female**	**Female to Male**	**Female to Female**
N	22	17	24	22
Transfer in Percent	16.34	18.24	34.58	26.82
Age (in years)	22.82	22.65	21.86	22.33
Income level	1.82	1.82	1.54	1.59
Single in Percent	81.82	82.35	75	72.73
Game theory knowledge in Percent	31.82	41.18	16.67	36.36
Donation history in Percent	59.09	76.47	54.17	45.45
**Take**	**Male to Male**	**Male to Female**	**Female to Male**	**Female to Female**
N	22	18	19	19
Transfer in Percent	23.64	33.33	40	47.37
Age (in years)	22.73	22.38	24.53	22.58
Income level	1.64	1.78	1.79	1.74
Single in Percent	63.64	77.78	57.59	73.68
Game theory knowledge in Percent	45.45	27.78	31.58	15.79
Donation history in Percent	63.64	38.89	57.89	68.42
