# Supplementary material for: Social preferences under chronic stress
Source: PLoS One. 2018 Jul 18;13(7):e0199528. doi: 10.1371/journal.pone.0199528 (PMC6051590; doi:10.1371/journal.pone.0199528)
Supplement: S2 Table — We tested reliability for the TICS summed value, as well as for the original screening scale for chronic stress (SSCS) and the nine facets of stress determined in the questionnaire: pressure to be successful (ERDR), lack of social recognition (MANG), chronic anxiety (SORG), excessive social demand (SOUE), social isolation (SOZI), social tensions (SOZS), excessive workload (UEBE), mental overload at work (UEFO), and dissatisfaction at work (UNZU). (PDF) [file pone.0199528.s005.pdf]

S2 Table Modified TICS is highly reliable

Reliability Analysis for the Original and Modified TICS

Scale Label	Number of Items	Cronbach's Alpha Original Version (3 months)	Cronbach's Alpha Modified Version (1 month)
ERDR	9	.90	.83
MANG	4	.84	.83
SORG	4	.88	.87
SOUE	6	.84	.85
SOZI	6	.88	.90
SOZS	6	.87	.86
SSCS	12	.91	.91
UEBE	8	.90	.92
UEFO	6	.87	.87
UNZU	8	.85	.83
Average	10	**.87**	**.87**
**TICS**	57	-	**.95**

Cronbach's Alpha values for the original version are taken from the TICS manual (Schulz et al., 2004)

TICS Descriptives

Mean	Minimum	Maximum	Median	25th Percentile	75th Percentile	SD	N
80.84	1	196	80.00	59.00	100.00	29.98	348
