# Supplementary material for: Social preferences under chronic stress
Source: PLoS One. 2018 Jul 18;13(7):e0199528. doi: 10.1371/journal.pone.0199528 (PMC6051590; doi:10.1371/journal.pone.0199528)
Supplement: S3 Table — (PDF) [file pone.0199528.s006.pdf]

S3 Table Relation Chronic Stress & Transfers in incentive compatible task

Model	(1)	(2)	(3)	(4)	(5)
	p_100	p_100	p_100	p_100	p_100
TICS	-0.084	-0.102	-0.251*	-0.042	-0.024
	(-0.83)	(-0.98)	(-1.79)	(-0.28)	(-0.17)
Gender Sender (0 = Male)		11.99**	-15.62	11.49*	5.124
		(1.98)	(-0.87)	(1.77)	(0.81)
Gender Recipient (0 = Male)		4.142	3.015	4.379	4.181
		(0.69)	(0.50)	(0.72)	(0.72)
Treatment (0 = Take)		0.108	0.982	0.654	-0.841
		(0.02)	(0.16)	(0.11)	(-0.14)
Gender Sender x TICS			0.343		
			(1.63)		
Acute Stress (scale)				0.101	0.000
				(0.74)	(0.00)
Anxiety (scale)				-0.285	0.009
				(-0.24)	(0.01)
Depression (scale)				-0.961	-1.024
				(-0.73)	(-0.81)
Age in years					0.610
					(0.55)
Game theory knowledge (0 = No)					22.95****
					(-3.67)
Single (0 = No, 1 = Yes)					2.897
					(0.45)
Income level (scale)					0.326
					(0.06)
Donations (0 = No, 1 = Yes)					9.722
					(1.59)
Constant	15.05*	8.492	20.01*	6.323	-0.895
	(1.73)	(0.86)	(1.67)	(0.63)	(-0.03)
Sigma					
Constant	37.99****	37.56****	37.25****	37.50****	34.87****
	(12.52)	(12.53)	(12.54)	(12.47)	(12.55)
Observations	185	185	185	183	181

There is no significant Relation between chronic stress and dictator game transfers

in an incentive compatible task; this result is robust to further experimental manipulations

Model	(1) p_100	(2) p_100	(3) p_100	(4) p_100	(5) p_100	(6) p_100	(7) p_100	(8) p_100	(9) p_100	(10) p_100	(11) p_100
TICS	-0.206**	-0.187*	-0.175	-0.176	-0.198*	-0.270**	-0.269	-0.259	-0.257**	-0.304	-0.252*
	(-2.04)	(-1.96)	(-1.21)	(-1.06)	(-1.74)	(-2.14)	(-1.13)	(-1.20)	(-2.07)	(-1.27)	(-1.76)
Gender S. (0 = Male)		23.02****	24.63			24.60****			24.78****		
		(4.21)	(1.53)			(4.32)			(4.36)		
Gender R. (0 = Male)		2.779	2.810	8.277	-1.248	1.471	6.532	15.56*	1.146	4.937	-3.445
		(0.52)	(0.52)	(0.91)	(-0.19)	(0.27)	(0.68)	(1.74)	(0.21)	(0.53)	(-0.52)
Treatment (0 = Take)		-15.06***	-15.01***	-20.61**	-11.42*	-14.60***	-20.83**	-7.068	-15.30***	-23.05**	-12.98*
		(-2.76)	(-2.74)	(-2.23)	(-1.69)	(-2.65)	(-2.13)	(-0.78)	(-2.80)	(-2.37)	(-1.84)
Gender Sende xTICS			-0.0202								
			(-0.11)								
Acute Stress						-0.083	-0.02	0.489**	-0.078	-0.061	-0.141
						(-0.73)	(-0.10)	(2.14)	(-0.69)	(-0.30)	(-1.00)
Anxiety						-0.195	0.760	-0.0525	-0.424	0.265	-0.839
						(-0.19)	(0.40)	(-0.03)	(-0.42)	(0.14)	(-0.70)
Depression						1.614	0.370	-1.160	1.380	0.355	1.797
						(1.32)	(0.18)	(-0.63)	(1.13)	(0.17)	(1.11)
Age									0.548	2.649	-1.040
									(0.52)	(1.45)	(-0.77)
Game theory knowl.									-5.723	-7.761	-5.029
									(-0.96)	(-0.76)	(-0.66)
Single									11.92*	17.52	8.528
									(1.93)	(1.55)	(1.16)
Income level									0.709	2.790	-0.706
									(0.13)	(0.28)	(-0.11)
Donations									-6.240	-8.858	-1.633
									(-1.16)	(-0.95)	(-0.24)
Constant	40.15****	33.32****	32.39**	30.71**	57.61****	36.55****	32.77**	11.49	21.25	-28.67	88.25**
	(4.66)	(3.80)	(2.60)	(2.06)	(5.65)	(4.00)	(2.02)	(0.85)	(0.72)	(-0.59)	(2.27)
sigma											
Constant	35.35****	32.62****	32.61****	37.15****	29.31****	32.44****	37.46****	36.91****	31.79****	36.04****	28.35****
	(13.31)	(13.38)	(13.37)	(8.37)	(10.46)	(13.31)	(8.24)	(8.24)	(13.32)	(8.28)	(10.47)
Observations	163	163	163	79	84	162	78	93	162	78	84

t statistics in parentheses; * $p < 0.10$, ** $p < 0.05$, *** $p < 0.01$, **** $p < 0.001$

8

9
